# Multiplexed Electrochemical Immunosensors for Clinical Biomarkers

**DOI:** 10.3390/s17050965

**Published:** 2017-04-27

**Authors:** Paloma Yáñez-Sedeño, Susana Campuzano, José M. Pingarrón

**Affiliations:** Departamento de Química Analítica, Facultad de CC. Químicas, Universidad Complutense de Madrid, E-28040 Madrid, Spain; susanacr@quim.ucm.es (S.C.); pingarro@quim.ucm.es (J.M.P.)

**Keywords:** multiplexed electrochemical immunosensors, biomarkers, cancer, cardiovascular, microfluidic, paper electrodes

## Abstract

Management and prognosis of disease requires the accurate determination of specific biomarkers indicative of normal or disease-related biological processes or responses to therapy. Moreover since multiple determinations of biomarkers have demonstrated to provide more accurate information than individual determinations to assist the clinician in prognosis and diagnosis, the detection of several clinical biomarkers by using the same analytical device hold enormous potential for early detection and personalized therapy and will simplify the diagnosis providing more information in less time. In this field, electrochemical immunosensors have demonstrated to offer interesting alternatives against conventional strategies due to their simplicity, fast response, low cost, high sensitivity and compatibility with multiplexed determination, microfabrication technology and decentralized determinations, features which made them very attractive for integration in point-of-care (POC) devices. Therefore, in this review, the relevance and current challenges of multiplexed determination of clinical biomarkers are briefly introduced, and an overview of the electrochemical immunosensing platforms developed so far for this purpose is given in order to demonstrate the great potential of these methodologies. After highlighting the main features of the selected examples, the unsolved challenges and future directions in this field are also briefly discussed.

## 1. Introduction

Most diseases have more than one marker associated with their incidence. Although a single biomarker can be used to diagnose a health disorder, there are issues in which this cannot accurately predict their occurrence. Measuring panels of biomarkers offers a more powerful approach to early detection of diseases and monitoring the responses of patients to therapy [1]. Moreover, the simultaneous determination of several biomarkers can minimize false negatives and false positives in clinical diagnoses more prone to occur when measuring a single molecule. To address this multianalyte detection, progress has been made to develop multiplexed biosensors capable of detecting simultaneously various targets then providing more accurate data for diagnosis and monitoring. In fact, in the last years, the availability of multiplexed biosensors has emerged as a valuable tool for detection and prognosis of some diseases, especially cancer.

Despite their sensitivity and commercial availability, conventional protein detection methods such as enzyme-linked immunosorbent assays (ELISAs), often take many hours to complete and usually only apply to one protein at a time. Therefore, more rapid, multiplexed methods also compatible with 7portable instrumentation are needed for point-of-care (POC) and surgical applications to improve the early and reliable personalized clinical diagnosis and thus the therapy efficiency. In this sense, electrochemical biosensors are characterized by inherent properties such as high sensitivity, portability, ease to use, simplicity of signals recording, and cost-effectiveness [2]. These advantages extend also to multiplexed systems [3] which are uniquely positioned to provide faster, point of care (POC) devices to detect biomarkers for clinical diagnosis [4]. This type of biosensor technology relies on multiple transductors and/or detection tags that act independently for each specific analyte [5]. In the particular case of multiplexed electrochemical immunosensors, they possess additional advantages related with improved detection efficiency, with detection limits reaching levels as low as units of femtograms per milliliter [6], reduced sample volume, versatility, and reliability, providing, in addition, good precision and reproducibility [7] and short time for analysis [8]. Electrochemical detection of panels of multiple proteins constitutes a suitable approach to early and more reliable detection of diseases and translation to personalized diagnostics [9]. However, more investigation is required since solutions to increase the number of analytes measured using a single device and a small sample volume of untreated complex samples (i.e., a drop of whole blood or tissue lysate), are highly demanded [4].

The construction of multilabeled immunoprobe platforms implies the need for discrimination between the signals corresponding to each analyte from the multiple antigen-antibody reactions [10]. This objective can be achieved by designing platforms through two different approaches: using multielectrode arrays where each immunoreagent is attached to each electrode, or by means of barcode configurations involving a unique electrode platform and different electroactive labels with dissimilar electrochemical properties for each analyte. Multi-electrode arrays need more complex electrochemical instrumentation since they usually consist of several sensing surfaces provided with individual reference and auxiliary electrodes and an independent n-channel electrochemical workstation. As advantages, these configurations use a single label, and “cross-talk” between the adjacent transduction elements is prevented [11]. On the other hand, the barcode approach makes use of distinct electroactive labels capable of generating appropriate and distinguishable signals at different potentials. A distinctive advantage is that the electrochemical responses can be monitored in a single amperometric or voltammetric scan [12,13,14]. However, “cross-talk” is a potential drawback if the detection potentials of the different redox tags are not sufficiently separated.

In both cases, solid carbon or metal electrodes usually modified with highly conductive substrates such as graphene or carbon nanotubes, and/or nanostructured with materials showing high ability for biomolecules adsorption (gold nanoparticles (AuNPs), chitosan) are used for immobilization of immunoreagents. In addition, as it will be shown below, some configurations of multiplexed electrochemical immunosensors involve the use of magnetic particles where the immunoreagent and signal tags for each analyte are immobilized outside the electrode. Furthermore, singular configurations of current great interest such as those using easily accessible and low-cost paper electrodes (PWEs) [15], and microfluidic systems [16,17] should be mentioned.

The most important features of recently reported multiplexed immunosensors for electrochemical detection of biomarkers have been summarized in Table 1 and Table 2. The main analytical characteristics of the methods developed for the simultaneous determination of biomarkers involving electrochemical immunosensors constructed with electrode arrays are given in Table 1 (refs. [7,8,18,19,20,21,22,23,24,25,26,27,28,29,30,31,32]). Since cancer is a major threat of global health, growing demand exists for the development of reliable devices for the detection of this disease, and therefore, most of the proposed designs are devoted to cancer markers. In addition, the relatively few designs described for the detection of cardiac biomarkers and other clinically relevant molecules are also considered. Furthermore, Table 2 summarizes the multiplexed immunosensors reported so far involving barcode configurations (refs. [6,12,13,14,33,34,35,36,37,38,39,40,41,42,43,44,45,46,47,48,49,50,51,52,53,54,55,56,57,58,59]). In the next sections we will focus not only on this type of biomarkers but also in those involved in cardiovascular diseases. Other clinically relevant species such as cytokines and hormones implied in inflammation processes and metabolic disorders are also reviewed. 

## 2. Multiplexed Electrochemical Immunosensors Involving Electrode Arrays

Electrochemical immunosensors constructed with electrode arrays (summarized in Table 1) are commonly fabricated with screen-printed electrodes (SPEs) containing two or more individual elements [60]. SPEs possess advantages derived not only from the good electrical properties, but also from the possibility to be fabricated with different materials and to be drawn in diverse geometries. Moreover, the small size of SPEs contributes to the great potential they have for POC tests, since allow sizing reduction of the electrochemical instrumentation down to small pocket-size devices. These disposable electrodes, characterized also by the low fabrication cost and the possibility for mass production [61], can be utilized with most of commercially available instruments using the most common electrochemical techniques: amperometry and differential pulse, or square wave voltammetry. Benchtop and portable instruments equipped with USB plugs, laptops or palm devices provided by various companies (PalmSens, DropSens and others) can be used for specific applications of immunosensors constructed with SPEs interfaced with personal computers or hand held devices such as smart phones. Furthermore, low-cost and friendly user platforms are currently available and easily customized, as well as coupled with electrochemical multi-arrays. 

**Figure 1:**
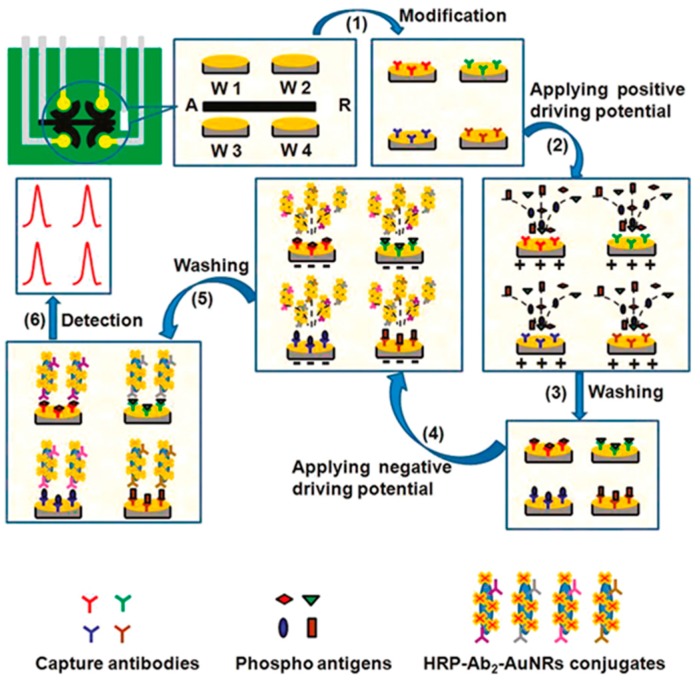
Multiplexed electrochemical immunoplatform for the simultaneous determination of phosphorylated p53 at Ser392 (phospho-p53_392_), Ser15 (phospho-p53_15_), Ser46 (phospho-p53_46_), and total p53 using electric field-driving and multi-enzyme labeling amplification. Reprinted from Ref. [8] with permission.

Most of the immunoassays used for multiplexed analysis are sandwich-type, frequently involving carrier tags attached to the secondary antibody. These labels fulfill the mission of amplifying the electrochemical response then providing a high sensitivity. A representative example of such configurations is the electrochemical immunoassay, integrating an enzyme amplification strategy, developed for the simultaneous determination of phosphorylated p53 at Ser392 (phospho-p53_392_), Ser15 (phospho-p53_15_), Ser46 (phospho-p53_46_), and total p53 (Figure 1). The tumor suppressor p53 protein plays a critical role in regulating cell growth and DNA repair processes. However, once stimulated, the phosphorylation of p53 on several amino acid residues would be increased, which may directly result in tumor development [62]. Serine 15 in human p53 is specially phosphorylated by ionizing radiation and UV which may damage the DNA repair process [63] whereas phosphorylation at serine 392 may be involved in human breast tumor and ovarian neoplasms [64]. Thus, these specific phosphorylation sites of p53 are useful biomarkers of diseases. The multiplexed platform was constructed by immobilization of the different capture antibodies on each of four working electrodes. Moreover, gold nanorods were used as nanocarriers for co-immobilization of HRP and detection antibodies. An interesting characteristic of this design is the application of electric-field driving to accelerate the transport of the charged antigens and HRP conjugates to the electrode surface. The electrochemical responses were obtained from the sandwich-type immunoreactions by the reduction of the oxidized thionine which is produced by HRP in the presence of H_2_O_2_. Detection limits ranged between 5 and 30 pM [8].

A multiplexing electrochemical immunosensor was developed for the detection of prostate specific antigen (PSA) and interleukin 8 (IL-8) cancer related proteins by using a disposable screen-printed carbon electrode (SPCE) array with 16 elements as the detection platform, and a multi-labeled nanoprobe prepared by loading HRP and goat anti-rabbit IgG onto multiwalled carbon nanotubes (MWCNTs). The large number of HRP molecules on the carbon nanotubes allowed a high sensitivity to be obtained with LOD values of a few pg·mL^−1^ for both analytes [18] PSA was also determined along with prostate specific membrane antigen (PSMA) and interleukin-6 (IL-6) using a flexible polydimethylsiloxane slice deposited with 8 × 8 nano-Au film electrodes. Primary antibodies were linked with magnetic beads (Ab_1_-MBs) and immobilized on nano-Au film electrodes by application of a magnetic field. The secondary antibodies were conjugated with HRP and AuNPs and, upon the sandwich immunoassay implementation, electrochemical signals were obtained from H_2_O_2_ reduction [19].

Metal nanoparticles have largely demonstrated their usefulness as electrocatalytic elements for improving the selectivity and sensitivity of electrochemical detections. In this context, mesoporous metallic structures possess unique properties due to the presence of interconnected hollow channels that facilitate mass transport and enhance electron conductivity. An illustrative example related with multiplexing applications involves the use of mesoporous platinum nanoparticles (MPtNPs) which exhibit high electrocatalytic activity toward H_2_O_2_ reduction. Using MPtNPs as non-enzymatic labels for the secondary antibodies, a sandwich-type immunosensor array was constructed for carbohydrate antigen 125 (CA125), carbohydrate antigen 153 CA153) and carcinoembryonic antigen (CEA) tumor markers by covalent immobilization of capture antibodies onto a triple graphene modified-SPCE. The electrochemical measurements were recorded by differential pulse voltammetry after addition of H_2_O_2_ providing cathodic peaks with a high sensitivity and reproducibility [20].

**Figure 2:**
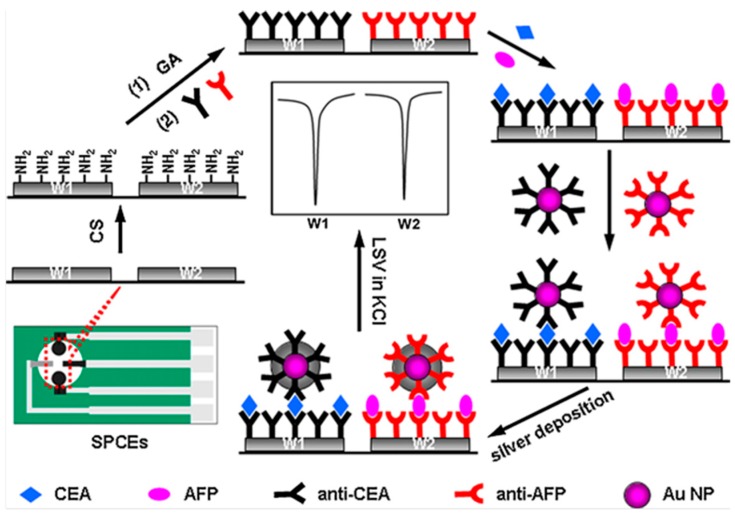
Scheme of the preparation and functioning of a multiplexed immunosensor for the determination of CEA and AFP involving stripping voltammetry of Ag catalytically deposited by gold nanolabels. Reprinted from Ref. [21] with permission.

Various multiplexed configurations imply stripping analysis of silver nanoparticles (AgNPs) which are characterized by a low oxidation potential and facile stripping conditions which make them easily detected. However, few works using AgNPs tags have been reported due to their limited stability in saline buffer and the tedious synthetic procedure [65]. An example is the immunosensor array constructed for the determination of CEA and α-fetoprotein (AFP) by immobilizing the capture antibodies onto chitosan modified SPCEs and preparation of a sandwich-type configuration with AuNPs-functionalized secondary antibodies (Figure 2). The electrochemical detection was carried out through silver deposition induced onto AuNPs and measuring silver re-dissolution currents by anodic stripping voltammetry. A deposition time of 4 min under dark was selected for silver followed by rinsing with water and linear sweep voltammetry in 1.0 M KCl solution. The peak at positive potential (+0.04 V approximately) at each working electrode of a dual SPCE allowed detection of both antigens simultaneously with no interference from dissolved oxygen [21]. Same authors prepared a similar immunosensor in which CEA and AFP capture antibodies were immobilized in the same manner and trace tags consisting of streptavidin-functionalized AgNPs/MWCNTs conjugates were used for further linkage of biotinylated detection antibodies. Through a sandwich-type immunoreaction and additional silver deposition, the stripping responses provided a high sensitivity with detection limits down to 0.093 (CEA) and 0.061 (AFP) pg·mL^−1^ [22].

Another interesting configuration is that reported by Ge et al. [23] where a multiplexed immunosensor array composed by three screen-printed graphite electrodes modified with graphene and AuNPs was employed for immobilizing the capture antibodies for CA153, CA125 and CEA. Sandwich-type immunoassays were implemented on each electrode using alkaline phosphatase (AP)-labeled secondary antibodies functionalized onto a graphene/gold cluster, and 3-indoxyl phosphate (3-IP) as the enzyme substrate. In the presence of silver ions, the hydrolysis of 3-IP catalyzed by AP produced an indoxyl intermediate able to provoke silver metal deposition. The stripping responses measured by linear sweep voltammetry were linearly related to the logarithm value of each biomarker in a wide concentration range (see Table 1). Bioconjugates of streptavidin onto carbon nanohorns modified with AuNPs (Strept-AuNPs-CNHs) were also used as signal tags to induce silver enhancement for signal amplification. The immunosensor array was prepared on disposable SPCEs and applied to the determination of AFP and CEA tumor markers. Through a sandwich-type immunoreaction and biotin-streptavidin affinity reaction, the tags were captured on the immunoconjugates and silver deposition was induced. This method provided LOD values of 0.024 pg·mL^−1^ and 0.032 pg·mL^−1^ for AFP and CEA, respectively [7].

Estrogens exhibit an interesting tissue selective action of great biomedical importance in searching the optimal therapeutics for prevention and treatment of breast cancer and other diseases [66,67]. Research involving estrogen and progesterone receptors (ER and PR) has helped to implement them as key targets in the treatment of tumors, providing insights into the mechanism for their activation and inhibition [68]. Clinical data indicated that the ER+/PR- breast tumors are less responsive to endocrine therapy than ER+/PR+ tumors [69]. Therefore, the levels of both receptors have a great interest as a prognostic and predictive factor, becoming this determination a standard practice in the management of this neoplasm. With this objective, a multiplexed magneto-immunosensor using dual SPCEs was implemented for ERα and PR determination in cell lysates. Carboxylated magnetic microbeads were used for immobilization of the respective antibodies and sandwich-type immunoassays involving biotinylated antibodies and streptavidin-HRP conjugates were performed. After magnetic capturing of the immunocomplexes onto the electrode surfaces, amperometric detection was carried out upon addition of H_2_O_2_ in the presence of hydroquinone. This multiplexed immunosensor was successfully validated by discrimination of raw lysate of two types of metastatic breast cancer cells (MCF-7 and MDA-MB-436) with significantly different ERα/PR expression levels [24].

**Figure 3:**
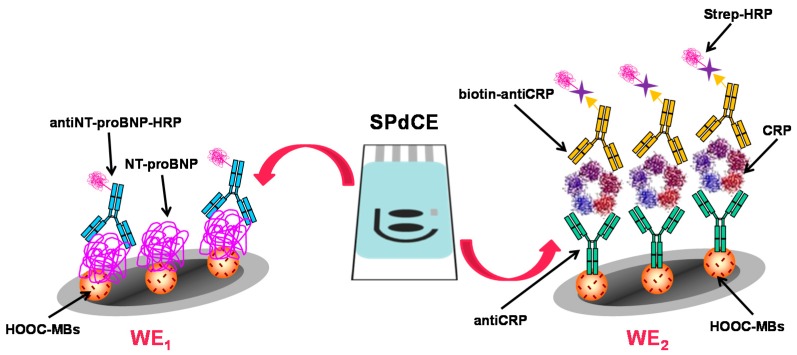
Schematic display of the disposable dual magnetoimmunosensor prepared for the determination of NT-proBNP and CRP. Reprinted from Ref. [26] with permission.

As Table 1 shows, multiplexed immunosensors for the determination of cardiac markers are still very scarce. Among the various protein biomarkers for monitoring of heart diseases, cardiac troponins (cTnT, cTnI) or myoglobin (Mb) concentrations in serum remain elevated after the occurrence of an acute myocardial infarction (AMI), while early markers such as cardiac reactive protein (CRP) and both B-type natriuretic peptide (BNP) and N-terminal ProBNP show rising concentrations on the occurrence of symptoms of systemic inflammation. Both types of biomarkers have significant value in diagnosis and prognosis of cardiac disease [25,70]. In an interesting paper, Esteban Fernández de Ávila et al. [26] proposed the first multiplexed configuration for NT-proBNP and CRP consisting of a magnetic microparticles-based immunosensing platform involving carboxylic acid-modified magnetic beads for covalent immobilization of NT-proBNP antigen anti-CRP specific capture antibodies (Figure 3). The quantification of both analytes was performed by indirect competitive and sandwich-type immunoassays, respectively, using HRP-labeled tracers. The electrochemical detection at dual SPCEs allowed the achievement of simultaneous independent amperometric readout for each cardiac biomarker. The developed methodology achieved very low detection limits of 0.47 ng·mL^−1^ for both analytes. The usefulness of the magnetoimmunosensor was evaluated by analysis of an international standard for CRP serum spiked with NT-proBNP. Importantly, this method allowed matching the clinically relevant concentration ranges for both cardiac biomarkers using the same electrode platform, and the whole multiplexed immunoassay could be completed in 1 h approximately.

Integration of sensors into a lab-on-a-chip offers advantages such as low cost through wafer-scale fabrication, simple electrochemical detection, low sample volume and multiplexing capability, along with improved sensitivity and specificity. A representative example of this strategy was recently reported by Gupta et al. [25]. They prepared an immunosensing array configuration consisting of a multielectrode sensor chip containing nine identical, electrically isolated, microelectrodes arranged in a 3 × 3 design where the specific immunoreagents were immobilized. The resulting immunosensor was applied to the simultaneous determination of CRP, cTnI and Mb by immobilization of the respective antibodies using carbodiimide chemistry. Differential pulse voltammetry was used to measure the decreases in current responses from ferrocyanide solutions as increased the concentration of each antigen. The usefulness of the developed device was evaluated by measuring complex protein mixtures prepared at concentration levels ranging between 0.05 and 5 μg·mL^−1^ (CRP and Mb), and from 0.02 to 2 μg·mL^−1^ (cTnI). Another immunosensor for direct analysis of fingerprick blood was prepared with an integrated chip and applied to the simultaneous determination of three cardiac biomarkers: cTnT, creatine kinase MM (CK-MM), and creatine kinase MB (CK-MB). A silicon nanowire (SiNW) array sensor chip and a filtration chip for plasma separation from blood were used. The specific antibodies were immobilized onto SiNW chips after treatment with APTES and glutaraldehyde, and measurements were obtained by the resistance changes caused by the specific binding, calculated from gradient of the current-voltage curves registered with the chip immersed in PBS buffer solutions in the absence and in presence of each antigen. A low detection limit of 1 pg·mL^−1^ for the three cardiac biomarkers from 2 μL blood was attained in 45 min [27].

Cytokines are proteins of low molecular weight strongly associated with the immuno system [71]. They play critical roles in control of cell replication, apoptosis, chemically-induced tissue damage repair, inflammatory processes, and also in cancer development and progression [72]. Due to their usefulness as biomarkers in numerous diseases, the design of immunosensors for the determination of these proteins is currently of great interest. However, there are virtually no immunosensors for their simultaneous determination. In a recent article, an electrochemical platform for the simultaneous detection of the cytokines interleukin-1β (IL-β1) and factor necrosis tumor α (TNF-α) was proposed. IL-1β is a pro-inflammatory cytokine involved in immune processes and chronic neurodegenerative diseases such as Alzheimer [73]. Furthermore, the role of TNF-α is critical in many diseases including rheumatoid arthritis [74] or cancer [75]. Biological activities of both cytokines have been reported to be synergistic and overlapping. For example, these proteins are considered good biomarkers to predict side effects of cancer therapy such as oral mucositis [76] and for the detection of periodontal diseases [77]. With the aim to perform the simultaneous determination of TNF-α and IL-1β, a multiplexed electrochemical immunosensor using dual SPCEs modified with 4-carboxyphenyl-functionalized double-walled carbon nanotubes (HOOC-Phe- DWCNTs) was constructed. Capture antibodies were immobilized onto HOOC-Phe-DWCNTs/SPCEs in an oriented form making using of the commercial polymeric coating Mix&Go™. Sandwich type immunoassays were implemented with amperometric signal amplification through the use of poly-HRP streptavidin conjugates. Calibration plots with ranges of linearity extending between 0.5 and 100 pg·mL^−1^ and from 1 to 200 pg·mL^−1^ for IL-1β and TNF-α, respectively, were obtained with limits of detection of 0.38 pg·mL^−1^ (IL-1β) and 0.85 pg·mL^−1^ (TNF-α). The dual immunosensor was validated by application to the simultaneous determination of both cytokines in human serum spiked at clinically relevant concentration levels and in real saliva samples [28]. Related with cytokines determination, Baraket et al. [29] prepared an electrochemical immunosensor using silicon chip technology for real time detection of various interleukins: IL-1β, IL-10, and IL-6 which were secreted in acute stages of inflammation. The chip was fabricated with eight gold microelectrodes through electrically addressable diazonium-functionalized antibodies and monitoring of the immunocomplexes formation by electrochemical impedance spectroscopy (EIS) which allowed the simultaneous detection of the analytes in a 1–15 pg·mL^−1^ concentration range.

**Figure 4:**
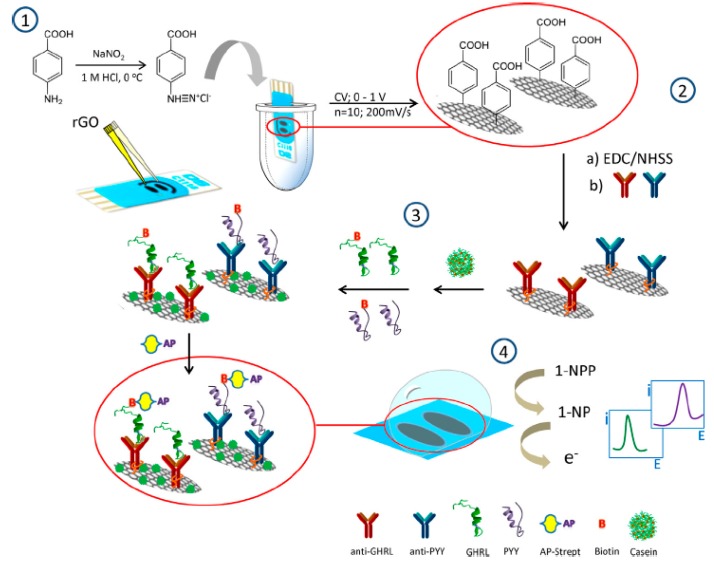
Scheme of the different steps involved in the preparation and functioning of the dual GHRL and PYY immunosensors. Reprinted from Ref. [30] with permission.

Peptide YY (PYY) is a gut hormone primarily released from endocrine cells of the distal digestive tract which plays an important role in regulating food intake and energy balance, and is related to diabetes type 2 and insulin resistance [78]. Ghrelin (GHRL) is an acylated peptide secreted in the stomach with growth-hormone-releasing function that acts as a stimulant of motor activity and appetite suppression (orexigen effect) in the regulation of energy homeostasis [79]. Concentrations of these hormones have been found to be elevated in anorexic patients, while lower levels appear in obese subjects. Taking into account the interest of knowing the amount of both PYY and GHRL in biological fluids, a method for the simultaneous determination of both hormones in human serum and saliva was implemented by means of an electrochemical immunosensor in which dual screen-printed carbon electrodes modified with reduced graphene oxide (rGO) were used as scaffolds for the immobilization of the corresponding capture antibodies. Grafting diazonium salt of 4-amino-benzoic acid (4-ABA) on rGO/dual SPCEs was performed (Figure 4). This treatment, based on the covalent attachment of aryl radical onto the carbon surface provides compact and stable monolayers that can be further used to obtain specific functionalities [80]. Competitive immunoassays were employed and the electrochemical responses monitoring the affinity reactions were obtained by differential pulse voltammetry (DPV) upon addition of 1-naphthyl phosphate (1-NPP). Under the optimized working conditions, linear current vs. log [hormone] plots extending between 10^−3^ and 100 ng·mL^−1^ GHRL and from 10^−4^ to 10 ng·mL^−1^ PYY were obtained [30].

The diagnosis of Cushing’s syndrome [81] is one of the most challenging problems in clinical endocrinology. Different levels of adrenocorticotropin (ACTH) and cortisol concentrations can be found in serum or urine depending on the type of Cushing’s syndrome, primary adrenal tumors or hyperplasia. Therefore, the development of analytical methods able to provide multiplexed determination of both hormones should help to perform a faster classification of the patients as well as an easier diagnosis of this kind of diseases. With this objective, Moreno-Guzmán et al. [31] prepared a dual electrochemical immunosensor for the multiplexed determination of ACTH and cortisol. The strategy employed in this work involved the immobilization of the immunoreagents through the use of a boronic acid derivative which interacts with sugars to form cyclic boronate esters at room temperature [82]. This kind of binding reaction successfully achieved the orientation of antibody molecules onto the electrode surface [83]. The corresponding ACTH and cortisol antibodies were immobilized on aminophenylboronic acid-modified dual SPCEs and competitive immunoassays involving biotinylated ACTH and alkaline phosphatase labeled streptavidin, or alkaline phosphatase labeled cortisol were implemented. Calibration plots were obtained by DPV with ranges of linearity between 0.1–500 ng·mL^−1^ cortisol and 5.0 × 10^−5^–0.1 ng·mL^−1^ ACTH. Moreover, the usefulness of the dual immunosensor was demonstrated by analyzing certified human serum samples with good recoveries.

Celiac disease (CD) is a gluten-induced autoimmune enteropathy characterized by the presence of antibodies against gliadin (AGA) and anti-tissue transglutaminase (anti-tTG) which are the specific serological biomarkers of the disease. A disposable electrochemical immunosensor for the simultaneous detection of IgA and IgG type AGA and anti-tTG antibodies in real patient’s samples was prepared using dual SPCEs modified with MWCNTs and AuNPs. The immunosensing strategy implied the interaction of the respective human antibodies with gliadin and tTG specific antigens immobilized on the nanostructured electrode surface followed by addition of anti-H-IgA-AP or anti-H-IgG-AP, respectively, and a mixture solution of 3-indoxyl phosphate (3-IP) and AgNO_3_. Under these experimental conditions, and as commented above [60], the hydrolysis of 3-IP catalyzed by AP produced an indoxyl intermediate able to provoke silver metal deposition that allowed to develop an anodic stripping method for the sensitive determination of the antibodies [32].

## 3. Multiplexed Electrochemical Immunosensors Using Barcode Configurations 

As was commented in the Introduction section, the use of a single electrode for the preparation of multiplexed immunosensor configurations presents the relevant advantages of simplicity, lower cost, and the possibility of obtaining the various analytical responses in a single recording. Problems derived from the mutual interference or overlapping of the electrochemical signals (“cross-reactivity”) may be avoided using as labels redox probes with differentiate electrochemical behavior tagging the immunoconjugates prepared on the same electrode platform. As it will be shown below, in some cases, the developed configurations require the application of a physical barrier for separation in order to obtain independent responses for the analytes. As Table 2 shows, all the methods described so far have been developed for the simultaneous detection of multiple tumor markers. 

Prussian Blue (PB) [33,34,35], toluidine blue (TB) [33], ferrocene [34,36,37] or ferrocene carboxylic acid (Fca) [6,38] , thionine (THI) [6,12,35,36,37,38] and anthraquinone (AQ) [6], have been used as carrier tags and redox mediators to prepare immunoconjugates for distinguishable detection in various configurations. These model electroactive compounds have demonstrated to serve as compatible redox probes providing well-defined and independent electrochemical responses with sufficient peak potentials separation to carry out the detection of the analytical signals corresponding to each analyte biomarker. Since the electrochemical behavior of these redox species depends on the pH value, optimization of this variable is essential to attain improved selectivity. An illustrative example is the work reported by Chen et al. [33] who prepared an electrochemical immunosensor with a sandwich-format for the simultaneous determination of CEA and AFP using carboxylated graphene nanosheets conjugated with TB or PB and anti-CEA or anti-AFP secondary antibodies, respectively. The detection pH was optimized because of the influence of this variable not only on the current and potential values of each redox probe but also on the stability and activity of antibodies and antigens. In this example, a pH value of 6.5 was selected. The corresponding capture antibodies were immobilized onto a glassy carbon electrode modified with chitosan (Chit) and AuNPs. The simultaneous detection of CEA and AFP was performed within a linear range of 0.5–60 ng·mL^−1^ for both analytes by measuring the DPV reduction peak currents of the redox probes. These same authors prepared also a similar configuration for AFP and CEA using functionalized Chit composites with the capture antibodies immobilized in the same manner mentioned above. The signal tags were fabricated by immobilizing PB or Fc onto Chit following by absorbing AuNPs to immobilize labeled anti-AFP and anti-CEA, respectively. As in the previous work, sandwich-type immunoassays were employed for the simultaneous detection of AFP and CEA by measuring DPV electrochemical responses of the redox labels [34]. Compared with the previous paper [33], this latter strategy allowed obtaining calibration plots with wider linear ranges and slightly lower limits of detection for both biomarkers. These improvements were attributed to the use of Chit-AuNPs composites which combine the large amount of active sites for immobilizing capture antibodies with the high capacity of AuNPs for promotion of the electron transfer. A similar configuration was also proposed by Feng et al. [35] for the same analytes using AuNPs decorated-rGO-carried PB or THI as the label tags. Interestingly, capture antibodies were immobilized onto a GCE modified with a hierarchically aloe-like gold microstructure (HAG) which was prepared by electropolymerization of aniline onto an rGO/GCE followed by AuNPs deposition. This sensing interface provided good biocompatibility and large effective area which greatly enhanced the immobilization of primary antibodies and, through a sandwich-type immunoassay, allowed the simultaneous detection of CEA and AFP within a linear range of 0.6–80 ng·mL^−1^ by DPV measurement of the reduction currents of PB and THI, respectively.

**Figure 5:**
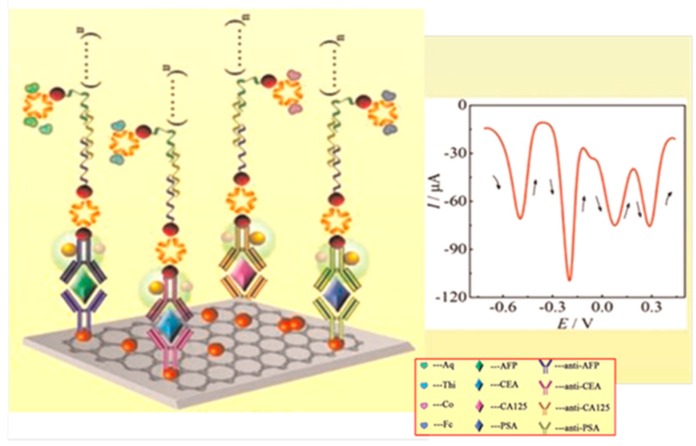
Schematic illustration of the fundamentals for the preparation of a sandwich-type immunosensor for the simultaneous detection of four cancer biomarkers, CEA, AFP, CA125 and PSA. Inset shows an example of the DPV recorded voltammogram. Modified from Ref. [6] with permission.

In an original and interesting configuration, four cancer biomarkers, CEA, AFP, CA125 and PSA were used as target compounds in the development of a multiplexed sandwich-type electrochemical immunosensor involving the immobilization of the corresponding antibodies onto a graphene/AuNPs hybrid film deposited onto a GCE. Biotinylated secondary antibodies (Biotin-Ab_2_) were immobilized by gold magnetic nanomaterials (AuNPs/SiO_2_-Fe_3_O_4_) and streptavidin was used to connect Biotin-Ab_2_ with biotinylated oligonucleotides chains added to trigger hybridization chain reaction so as to form long dsDNA containing abundant biotins and react with Strept-labeled redox probes. As Figure 5 shows, AQ, Fca, THI and Co(bpy)_3_^3+^ were the electroactive labels so that the resulting Ab_2_ bioconjugates, plenty of redox probes, could provide sensitive independent responses for the analytes. At the optimal experimental conditions, the immunosensor allowed the simultaneous determination of the four biomarkers in a wide range of concentrations, and provided very low limits of detection (see Table 2). Moreover, cross-reactivity studies revealed that deviations in DPV currents ranging between 1.5 and 2.2% were found for the determination of one biomarker in the presence of the others at concentrations up to 40 times higher [6].

The use of nanoparticles functionalized with each specific label tag constitutes a useful strategy for obtaining sensitive and selective responses for the detection of multiple biomarkers. For example, THI and Fca were incorporated onto mesoporous Fe_3_O_4_ NPs for the preparation of carrier tags for the simultaneous determination of squamous cell carcinoma associated antigen (SCC-Ag) and CEA. The magnetic nanomaterial can immobilize a high loading of electrochemical redox probes and secondary antibodies and promote the electron transfer, the resulting redox probes providing two well-separated peaks by DPV. An additional amplification of the electrochemical responses was obtained by using a conducting polymer, poly[3-(1,10-dimethyl-4-piperidino methylene)thiophene-2,5-diylchloride] (PDPMT-Cl), as a modifier of the electrode surface. This material is characterized by the good chemical and thermal stability together with the high biocompatibility and electrical conductivity. The resulting PDPMT-Cl/GCE was prepared by dropping a dispersion of the polymer in chitosan and used for immobilization of the capture antibodies. Sandwich immunoassays were performed by using secondary antibodies conjugated with the magnetic carrier tags and provided wide linear ranges with detection limits of 4 pg·mL^−1^ and 5 pg·mL^−1^ for SCC-Ag and CEA,respectively [38].

**Figure 6:**
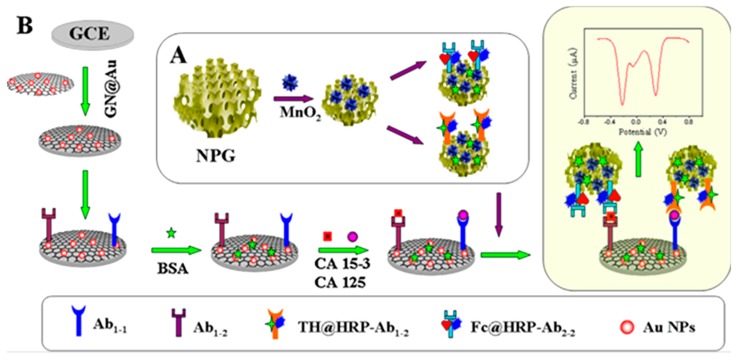
Schematic illustration of the fabrication procedure of the immunosensor developed for CA15-3 and CA125 using NPG-MnO2 and THI or Fc as the redox probes. (**A**) Functionalization of NPG with cactus-like MnO2; (**B**) Steps involved in the preparation of the immunosensor. Reprinted from Ref. [37] with permission.

In addition to the direct measurement of the redox probes responses, other immunosensor platforms take advantage of the electrocatalytic activity of such electroactive compounds toward the electrochemical reactions of H_2_O_2_ mediated by HRP. Signal variations related with such process have also been used for biomarkers quantification. For example, a multiplexed immunosensor with catalytic amplification was proposed using cactus-like MnO_2_ functionalized-nanoporous gold (NPG-MnO_2_) to immobilize HRP and THI or Fc as the redox probes for the simultaneous detection of CA15-3 and CA125, respectively (Figure 6). The synergy between the electrocatalytic effects of NPG and MnO_2_ nanoparticles yielded a highly conductive composite easily modified with secondary antibodies to serve as efficient signal tags with good bioactivity for clinical diagnosis [37].

An electrochemical immunosensor for simultaneous determination of CEA and AFP was prepared using THI and Fc covalently conjugated on anti-AFP and anti-CEA antibodies, respectively, to act as electrochemical tags. The resulting conjugates were co-immobilized on a AuNPs/GCE, and HRP was immobilized onto the modified electrode. Once the immunocomplexes were formed, the active center of the immobilized HRP was partially inhibited with the subsequent decrease in the enzyme activity for reduction of H_2_O_2_. This immunosensor enabled the simultaneous determination of AFP and CEA within a dynamic range between 0.01 and 50 ng·mL^-1^ and the same detection limit, 0.01 ng·mL^−1^, for both target compounds [36].

**Figure 7:**
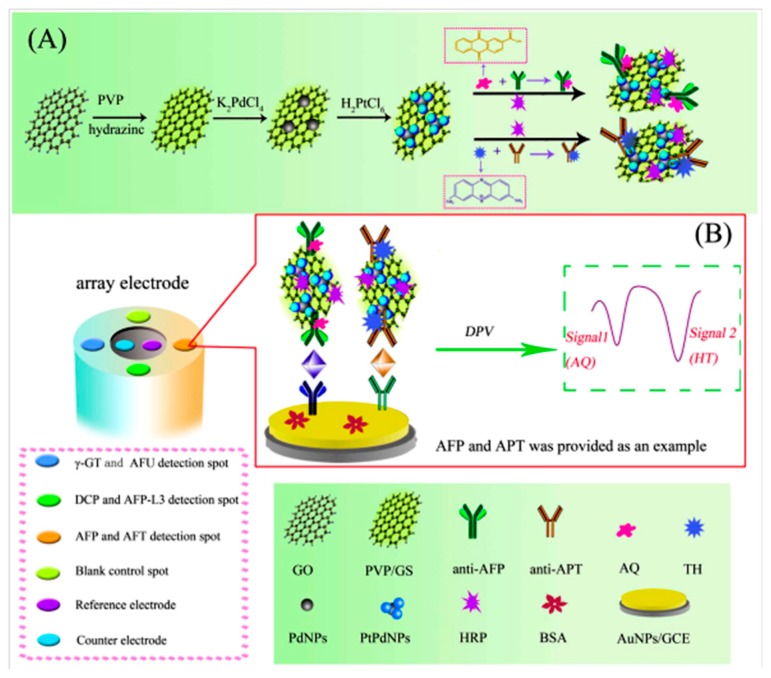
Preparation of HRP/PtPd/GS labeled redox probe branched antibodies (**A**). (**B**) Stepwise immunosensor fabrication process and DPV electrochemical responses. Reprinted from Ref. [12] with permission.

Yang et al. [12] prepared an original multiplexed immunosensor combining multi-label strategy and multi-spot assay with an array electrode for the simultaneous detection of six biomarkers for hepatocellular carcinoma (HCC). As Figure 7 shows, each electrode surface allowed the determination of two target proteins. The reference and counter electrodes are surrounded by four AuNPs/GCE working electrodes. AQ and THI were used for preparation of the carrier tags for the determination in pairs of AFP and APT (abnormal prothrombin), DCP (des-c-carboxyprothrombin) and AFP-L3 (*lens culinaris* (LCA)-reactive fraction of AFP), and γ-GT (γ-glutamyltranspeptidase) and AFU (α-L-fucosidase), by conjugation onto graphene nanosheets modified with Pt-Pd bimetallic nanoparticles. In the presence of H_2_O_2_, the electrocatalytic effect of both the electroactive compounds and the nanoparticles contributed to achieve well separated DPV responses with a high sensitivity.

Cyclodextrin−thionine−graphene (CD-THI-G) and cyclodextrin−ferrocene−graphene (CD-Fc-G) nanostructures were synthesized and employed for labeling of HRP-anti-AFP and HRP-anti-CEA conjugates, respectively. Using a sandwich-type assay format involving immobilization of the capture antibodies onto CD-G-modified GCE, two separate cathodic peaks corresponding to the catalytic effect of HRP toward the reduction of H_2_O_2_ in the presence of thionine and ferrocene were recorded. Under the optimal conditions, the multiplexed immunoassay enabled the simultaneous determination of CEA and AFP with wide working ranges of 0.001–60 ng·mL^−1^ AFP and 0.003–40 ng·mL^−1^ CEA, with detection limits of 0.5 and 0.8 pg·mL^−1^, respectively [39].

Electroactive compounds such as PB and THI can also be used as coatings of electrode surfaces to provide direct electrochemical responses which are dependent on biomolecules loading. This alternative can be employed for the preparation of multiplexed immunosensors if separate zones with a different coating each are fabricated. An example is the use of indium tin oxide (ITO) sheets as working electrodes for simultaneous determination of CEA and AFP.

Coatings of rGO/THI/AuNPs and rGO/PB/AuNPs onto the ITO surface were prepared with electrode slides separated lengthways into two uniform parts using insulation glue thus avoiding the cross-talk between the two portions. Anti-CEA and anti-AFP were immobilized onto the respective coatings, and the immunosensing detection was made in a single voltammogram by monitoring the decrease in the currents of PB and THI due to the formation of the antibody-antigen immuno complexes. This method enabled the simultaneous determination of CEA and AFP with linear working ranges of 0.01–300 ng·mL^−1^ [40]. A similar configuration was prepared with fluorine tin oxide (FTO) sheets coated with tryptophan and caffeic acid-based resin (TCCR) microspheres decorated with AuNPs and TB or PB. The resulting surface contained a lot of amino and hydroxy groups suitable as immobilization sites for the capture antibodies and facilitated the electron transfer. Simultaneous detection of CEA and neuron specific enolase (NSE) was performed by prepared two separated slides by means of an insulating glue, and measuring the decreasing in TB or PB corresponding currents caused by the immunocomplexes formation. Such decreases are directly related to each biomarker concentration within a linear range from 0.2 to 25 ng·mL^−1^ for both CEA and NSE [41].

**Figure 8:**
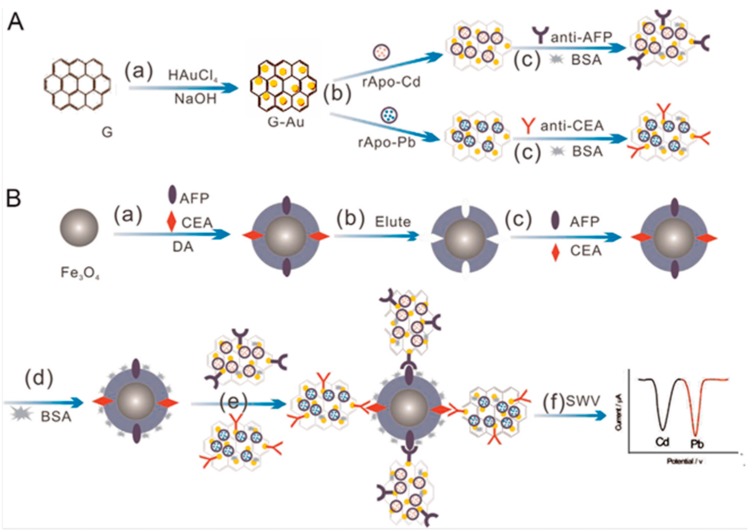
Scheme of the multiplexed electrochemical immunosensor for the simultaneous determination of AFP and CEA. Preparation of signal tags (**A**); preparation of MMIP and capture of AFP and CEA antigens followed by immunocomplexation and electrochemical detection (**B**). Reprinted from [43] with permission.

An electrochemical immunosensor for the simultaneous detection of phosphorylated proteins phospho-p53_15_ and phospho-p53_392_ was constructed using different apoferritin-templated metal phosphates: cadmium (ATCP) and lead (ATLP). These metallic phosphates were modified with SiO_2_ and AuNPs, and conjugated with the corresponding detection antibodies (Ab_21_ and Ab_22_) to be used as distinguishable signal reporters and detection sensitivity enhancers. Furthermore, magnetic Fe_3_O_4_ nanoparticles functionalized with phospho-p53_15_ and phospho-p53_392_ capture antibodies were also prepared and employed to specifically interact with the antigens to form sandwich-type immunoconjugates with the resulting ATCP/SiO_2_@Au/p53_15_ -Ab_21_ and ATLP/SiO_2_@Au/p53_392_-Ab_22_. The distinguishable current responses were obtained by electrochemical detection of cadmium and lead ions after dissolution with acid using square wave voltammetry (SWV). Linear relationships between the measured peak currents and the concentration of phospho-p53_15_ and phosphor-p53_392_ were obtained over the 0.1–20 ng·mL^−1^ and 0.05–20 ng·mL^−1^ ranges, respectively [42]. Another interesting work makes use of Cd^2+^ and Pb^2+^ apoferritins for the construction of an electrochemical multiplexed immunosensor for the simultaneous determination of AFP and CEA. ApoCd^2+^ and ApoPb^2+^ were immobilized onto graphene/AuNPs hybrids followed by the incorporation of the corresponding antibodies. Separately, dual-template magnetic molecularly imprinted polymers (MMIPs) were fabricated by coating Fe_3_O_4_ NPs with poly(dopamine) and used for capturing AFP and CEA antigens and further immunocomplexation with the respective graphene/AuNPs/ApoM^2+^-Ab conjugates (Figure 8). Using a bismuth film-deposited modified GCE, the electrochemical measurements were obtained in weakly acidic solutions by SW anodic stripping voltammetry of Cd and Pb after application of a deposition potential of −1.2 V vs. Ag/AgCl for 120 s. This method enabled the simultaneous determination of AFP and CEA in a single run with wide dynamic ranges of 0.001–5 ng·mL^−1^ [43].

B-cell lymphoma 2 (Bcl-2) and Bcl-2-associated X protein (Bax) are useful biomarkers to monitor de apoptosis of tumor cells. With the objective of obtaining simultaneous information of the levels of both species in biological samples, Zhou et al. [44] prepared recently a simple dual-signal-labeled electrochemical immunosensor involving the use of rGO as the substrate deposited onto a GCE to immobilize the antibodies for further capturing the target antigens. The signal probes used in this configuration were CdSeTe@CdS quantum dots (QDs) and Ag nanoclusters (AgNCs) incorporated to mesoporous silica nanospheres by means of poly(ethylene imine) (PEI) or poly- (diallyldimethylammonium chloride) (PDDA), on which the secondary anti-Bcl-2 and anti-Bax antibodies, respectively, were immobilized. After the antigens were sandwiched between both antibody conjugates, the electrode was immersed into an acidic solution in order to dissolve the probes and, after Hg^2+^ addition, a deposition potential of −1.1 V vs. SCE for 12 min was applied. SWV was used to record the stripping voltammetric scans where the oxidation peaks of Cd (−0.8 V) and Ag (+0.3 V) were employed to quantify the corresponding biomarkers. The large difference between the peak potentials indicated the absence of cross-reactivity between the probes. Moreover, a detection limit of 0.5 fmol was achieved for both proteins. Interestingly, the immunosensor was applied to investigate Bcl-2 and Bax expressions from nilotinib-treated chronic myeloid leukemia K562 cells, and to evaluate the drug effect through the Bax/Bcl-2 ratio.

**Figure 9:**
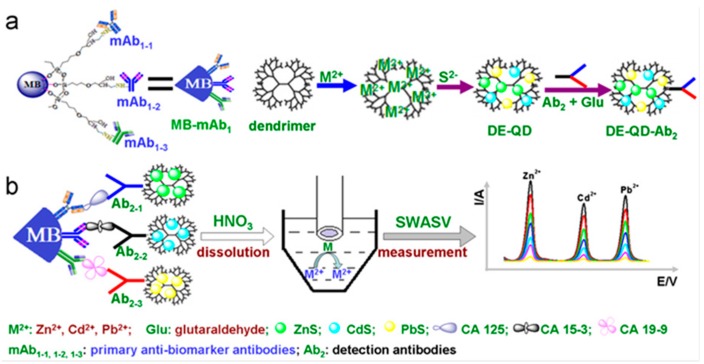
Multiplexed stripping voltammetric immunoassay using dendrimer-metal sulfide QD nanolabels (DE-QD) and tri-functionalized magnetic beads for the simultaneous determination of CA125, CA15-3 and CA19-9 cancer biomarkers: (**a**) preparation process and (**b**) measurement principle. Reprinted from [13] with permission.

Stripping voltammetry was also used for the simultaneous determination of CA125, CA15-3 and CA19-9 cancer biomarkers with an immunosensor fabricated with PAMAM dendrimer-metal sulfide quantum dots as the distinguishable signal tags. Tri-functionalized magnetic beads were employed as the support for immobilization of capture antibodies. The signal tags were prepared by precipitation of the corresponding sulfide into a mixture solution containing each metal and PAMAM, where the ions are coordinative linked to the amino groups of the dendrimer. The multiplexed determination of the analytes was performed by a sandwich-type immunoassay using anodic stripping voltammetry of cadmium, zinc, and lead upon these were released by acid from the nanolabels. A GCE modified with an in situ prepared mercury film was used to record the voltammograms. Figure 9 shows as the peak potentials appear sufficiently separated making the individual quantification in a mixture of biomarkers possible [13].

Ordered mesoporous carbon (OMC) is characterized by the high specific surface area and the large pore volume [84]. By incorporation of metals confined in the ordered matrix, the resulting materials (OMC-M) constitute useful structures with application as nanoprobes in electrochemical immunoassay. Fang et al. [45] prepared well-dispersed uniform metallic Zn and Cd nanocrystallites incorporated to OMC and the resulting OMC-Zn (or -Cd) were conjugated with the secondary antibodies and used as signal probes in the preparation of a multiplexed immunosensor for the determination of AFP and human epidermal growth factor receptor type-2 (HER-2). A GCE modified with MWCNTs and Chit was used as the electrode platform for immobilization of the capture antibodies and further interaction with the respective antigen. Sandwich configurations with the corresponding OMC-M conjugate were implemented and DPV was used for determining both antigens by measuring the oxidation peak currents. The method allowed low limits of detection of 0.6 and 0.35 pg·mL^−1^, for AFP and HER-2, respectively, to be obtained.

Voltammetic stripping analysis was also used for the determination of three cancer biomarkers, AFP, CEA and CA19-9 in a single run by employing CdS, PbS and Au nanoparticles to prepare the electrochemically distinguishable signal tags. Commercial Envision^™^ polymer containing numerous secondary antibodies and HRP was used for signal amplification after loading the detection antibodies and nanoparticles. The capture antibodies were immobilized onto a single magnetic bead and, once the immunocomplexes with the corresponding antigen were formed, sandwich-type immunoconjugates were prepared by interaction with the respective Envision^™^-NP-secondary antibody. A microcell provided by a glassy carbon microelectrode was used to carry out the voltammetric analysis. Anodic stripping by DPV after application of an accumulation potential of −1.2 V for 120 s was used for quantification of AFP and CEA upon dissolution of the corresponding CdS and PbS in acidic medium. Cathodic stripping by DPV was used to determine CA19-9 by application of a constant potential of +1.3 V for 30 s [46].

Another strategy for the preparation of signal tags with well-differentiated electrochemical behavior consists in the complexation of metallic ions to the modified surface of metal nanoparticles. For example, Cd^2+^ and Cu^2+^ complexed with amino-capped platinum porous nanoparticles (PtPNPs) were conjugated with anti-CEA and anti-AFP antibodies, respectively, and used as distinguishable signal tags for capturing antigens. An ionic-liquid-modified GCE was used as the electrode substrate for immobilization of capture antibodies and a sandwich-type immunoassay was implemented after complexation with the antigens and interaction with the as prepared signal tags. Voltammetric responses were recorded by DPV with no need for acid dissolution or application of any potential, and the peak heights of each voltammetric signal corresponded with the concentration of each antigen [14]. A similar method was proposed for the preparation of AuNPs-anti-AFP-Pb^2+^ or AuNPs-anti-CEA-Cu^2+^ labels with the ions linked to each conjugate by the amino groups of the protein. In this case, the capture antibodies were immobilized onto a GCE modified with Chit/AuNPs and glutaraldehyde, and sandwich-type immunoassays were performed for the determination of AFP and CEA. Interestingly, in this configuration, the potential values of the reduction peaks obtained by DPV exhibited a difference of 500 mV [47]. These same biomarkers were also determined with another immunosensor using MWCNTs/AuNPs-Ab_2_ with linked Cd^2+^ or Pb^2+^ as the electroactive labels. SWV was used for quantification of the analytes obtaining similar detection values than the previous method (see Table 2) but smaller peak separation for the voltammetric peaks [48].

Carbon nanospheres (CNSs) have demonstrated to be an excellent material for the immobilization of signal probes and biomolecules. Among its advantages, it is worth mentioning the presence of abundant reactive oxygen functional groups, the adsorption capability to organic and inorganic materials, and the biocompatibility. In addition, important properties of these particles such as their shape, size, and surface characteristics may be tuned by a careful control of the synthesis experimental conditions [85]. They are usually obtained by hydrothermal carbonization of carbohydrates by applying methods requiring long periods of time [86], but the duration of treatment can be shortened significantly using microwave assisted procedures. 

**Figure 10:**
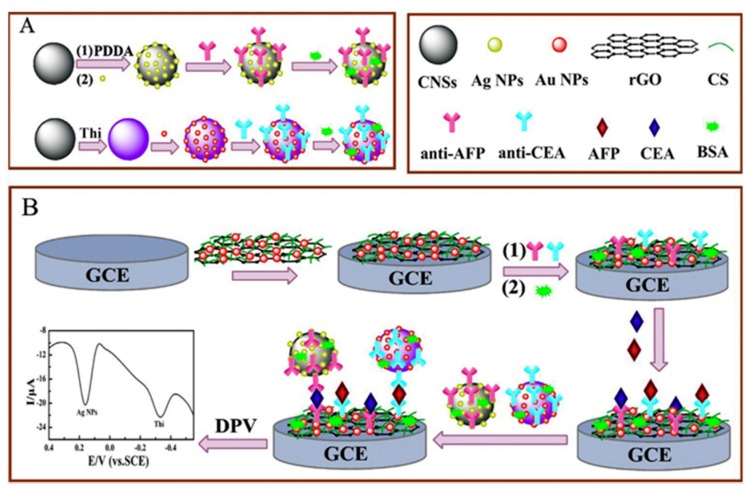
Multiplexed electrochemical immunosensors for detection of CEA and AFP using CNSs coated with AgNPs or AuNPs and THI for immobilization of secondary antibodies. Preparation of immunoprobes (**A**). Immunosensor fabrication procedure and recorded voltammetric signals (**B**). Reprinted from [49] with permission.

An interesting example of the application of this nanomaterial for the preparation of multiplexed electrochemical immunosensors is the use of CNSs coated with AgNPs or AuNPs and THI for immobilization of secondary antibodies, for detection of CEA and AFP. An electrode surface was prepared involving modification of a GCE with AuNPs/reduced graphene oxide (rGO) nanocomposites for assembling capture antibodies. In the presence of both proteins, two labels were attached onto the surface of the rGO/AuNPs nanocomposites via a sandwich immunoreaction. As Figure 10 shows, when recording DP voltammograms with the resulting immunosensor, two distinguishable peaks appeared at +0.16 V (corresponding to Ag NPs) and at −0.33 V (corresponding to THI). The great peak potential difference found between the peaks (close to 500 mV), demonstrated that both biomarkers could simultaneously detected in a single run [49].

A carbon gold nanocomposite (CGN) was prepared by a simple procedure through microwave-assisted carbonization of glucose to obtain CNSs and deposition of AuNPs. Taking advantage of the high adsorption ability of CGN toward organic compounds such as THI or 2,3-diaminophenazine (DAP) and metallic ions such as Cd^2+^, the corresponding hybrids were prepared and used to form immunoconjugates with anti-CEA, anti-PSA and anti-AFP secondary antibodies, respectively. Sandwich-type immunosensors for the simultaneous determination of the corresponding antigens were prepared by immobilizing the capture antibodies through electrostatic adsorption onto a GCE modified with a liquid ionic, graphene and poly(sodium-*p*-styrene sulfonate) (PSS). The respective immunoassays yielded three separate SW voltammetric signals. The resulting configuration allowed the determination of CEA, PSA and AFP in a single voltammetric scan with linear ranges between 0.01 and 100 ng·mL^−1^ and LOD values of 2.7, 4.8 and 3.1 pg·mL^−1^, respectively [50]. TiO_2_ nanoparticles have shown to be an efficient material for biosensor applications [87]. Besides the good biocompatibility, high reactivity and low cost, this nanomaterial is characterized by good conductivity, electrocatalytic activity [88], and the ability for immobilization of biomolecules. These properties were exploited by Wang et al. [51] to prepare a multiplexed immunosensor in which a nanohybrid composed of Nafion and TiO_2_ acted as a matrix for immobilization of immunoreagents and label tags. The antigens CA19-9 and CA15-3 were used as target compounds. Upon immobilization of the respective antibodies, the conjugates were labeled with methylene blue (MB) (CA19-9) or Co(dcbpy)_3_^2+^ complex (with dcbpy = 2,2’-bipyridine-4,4’-dicarboxylate) (CA15-3),. The resulting conjugates, anti-CA19-9-Nafion/TiO_2_/MB and anti-CA15-3-Nafion/TiO_2_/Co(dcbpy)_3_^2+^ were used to implement a sandwich-type immunoassay by means of the capture antibodies immobilized onto a gold electrode modified with gold nanoparticles. This immunosensor allowed a separation of the redox probes DPV peak potentials of 300 mV, allowing the determination of biomarkers at concentrations between 5 and 100 U·mL^−1^ (CA19-9), and from 1 to 100 U·mL^−1^ (CA15-5), with a good sensitivity for both proteins.

Polymer-nanoparticles composites have also been used for the preparation of multiplexed electrochemical immunosensors. These materials are of facile synthesis, and possess advantages such as the adjustable conductivity and proccessability. An interesting example is the synthesis of two types of redox-active polymer/AuNPs nanocomposites which were successfully used to fabricate a multiplexed electrochemical immunosensor for CEA and AFP biomarkers. Poly-(*o*-phenylene-diamine)(POPD)/AuNPs and poly(vinylferrocene-2-aminothiophenol) (poly(VFc-ATP))/AuNPs composites were synthesized at room temperature from the respective monomers using HAuCl_4_ as the oxidant. The resulting conjugated polymer/AuNPs showed an excellent redox-activity, good biocompatibility and stability. A simple immunoassay protocol was designed with the redox-active nanocomposites as the electroactive probes upon immobilization of anti-CEAandanti-AFP, and a gold electrode modified with poly(2-aminothiophenol) (PATP)used as the platform for immobilizing the capture antibodies. After implementing a sandwich-type immunoassay, DPV voltammograms were recorded and the independent pair of oxidation peaks characteristic of the polymers appearing at −0.43 V (PODP) and +0.2 V (VFc-ATP) were observed. The electrochemical immunosensor enabled the simultaneous monitoring of AFP and CEA in a wide linear range of 0.01–100 ng·mL^−1^ with detection limits of 0.006 ng·mL^−1^ for CEA and 0.003 ng·mL^−1^ for AFP [52]. 

**Figure 11:**
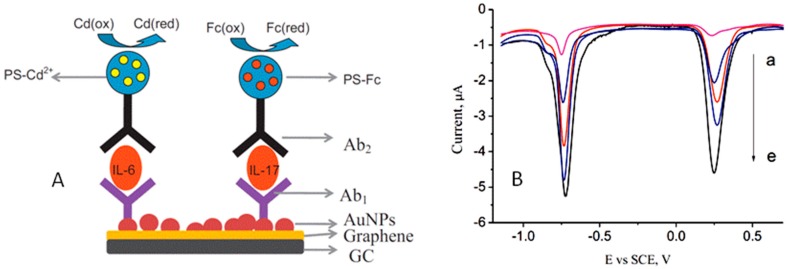
(**A**) Scheme of the electrochemical immunosensor constructed for the simultaneous determination of IL-6 and IL-17 using two different tags to polystyrene spheres and Cd^2+^ and Fc as the electroactive labels. (**B**) SW voltammograms recorded for the simultaneous detection of (a–e) 5, 50, 100, 500 and 1000 pg·mL^−1^ IL-6 (left) and IL-17 (right). Repinted from Ref. [53] with permission.

Interleukins 6 and 17 (IL-6 and IL-17) are cytokines secreted by T-cells acting as mediators in the stimulation of the immune response. They are involved in a variety of biological processes [89,90] including cancer diseases. There is evidence for IL-6 in the relationship with colorectal cancer and tumour necrosis [91] and also it has been found that IL-17 might be a pivotal cytokine involved in tumor progression of non-small cell lung cancer (NSCLC) [92]. A multiplexed electrochemical immunosensor was developed for ultrasensitive detection of both interleukins by immobilization of the specific detection antibodies and two different tags to polystyrene spheres (PSs). For this purpose, Cd^2+^ and Fc were selected as the electroactive labels, and the detection of biomarkers was performed by SWV after dissolving each PS conjugate with tetrahydrofuran (THF). Figure 11 shows as the immunosensing configuration provided voltammetric responses with a high peak potential difference. Moreover, the significant amount of electrochemical tags incorporated into PS greatly enhanced the sensitivity, with detection limits of of 0.5 and 1 pg·mL^−1^ for IL-6 and IL-17, respectively [53].

In addition to the immunosensors discussed so far, Table 2 summarizes analytical information for other multiplexed immunosensing designs using labels of graphene sheets tagged with THI, Co(bpy)_3_^3+^ and Fc redox probes for the determination of AFP, CEA and *streptococcussuis* serotype 2 (SS2) [54], or AuNPs-modified Prussian blue (or nickel hexacyanoferrates) for PSA and free PSA [55], as well as liposomes with encapsulated ascorbic acid (AA) and uric acid (AU) for monitoring neuron-specific enolase (NSE) and pro-gastrin-releasing peptide (ProGRP), two biomarkers for small cell lung cancer [56]. Other recent methods used alginates of Cd, Pb and Cd for the determination of AFP, CEA and PSA by DPV [57], or Cd and Pb apoferritins for the stripping voltammetric detection of AFP and CEA by SWV [58], and metal ions-doped chitosan poly(acrylic) nanospheres for determining four cancer biomarkers, CEA, CA19-9, CA125 and CA242, in a single voltammetric run [59].

## 4. Microfluidic Devices

Microfluidics has evolved as a powerful tool to be used in fundamental and applied biomedical research [93]. In 2005, Heinemann et al. [94] published one of the pioneer articles regarding the application of microfluidics in electrochemical immunoassay [16]. Since then, the advantageous properties of microfluidic devices such as the small working volumes, short assay times and high sensitivity has promoted the development of immunosensors coupled to the use of this emerging technology. In the particular case of multiplexed immunoassays, few methods have been published so far. A relevant example is the electrochemical system developed by Zhou et al. [95] for the simultaneous detection of cTnI and CRP cardiac markers using a poly-(dimethylsiloxane)-AuNPs composite microfluidic chip. In this method, CdTe and ZnSe QDs were conjugated with the different antibodies and, upon the implementation of the immunoassays, SW anodic stripping voltammetry at a carbon fiber microelectrode was used to detect the metal ions dissolved from the QDs in a microchannel via flow injection mode. 

A microfluidic electrochemical immunoassay was also reported for multiplexed detection of PSA and IL-6 in serum. It was based on the off-line capture of analytes by heavily-HRP labeled magnetic particles-antibody bioconjugates, and attachment of the capture antibodies to a chip with eight electrodes modified with glutathione-decorated AuNPs. Once the antibodies were immobilized onto the electrodes, a dispersion of the magnetic particles with the captured analytes was injected into the microfluidic chamber, and when it was filled, the flow was stopped during a time for incubation to allow that antigens were captured by the antibodies on the electrodes. After washing the system, a mixture of H_2_O_2_ and hydroquinone was injected into the microfluidic device and the amperometric responses were measured. The detection limits attained with this method were as low as 0.23 pg·mL^−1^ for PSA and 0.30 pg·mL^−1^ for IL-6 in diluted serum. Importantly, this immunosensor could measure both biomarkers in serum of prostate cancer patients in a total assay time of 1.15 h [16].

**Figure 12:**
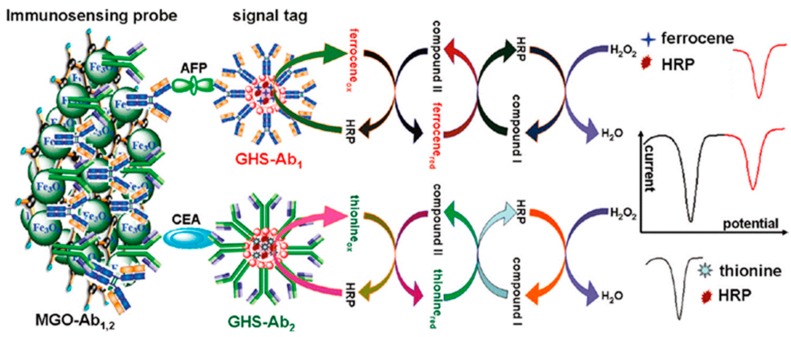
Scheme of the multiplexed electrochemical immunoassay for CEA and AFP using magnetic graphene nanosheets with immobilized capture antibodies as immunosensing probes, and multifunctional nanogold hollow microspheres (GHS) with encapsulated HRP-THI and HRP-Fc as distinguishable signal tags. Figure shows also some of the voltammograms recorded. Reprinted from Ref. [17] with permission.

A flow-through multiplexed immunoassay was designed for simultaneous electrochemical determination of CEA and AFP using magnetic graphene nanosheets with immobilized capture antibodies as immunosensing probes, and multifunctional nanogold hollow microspheres (GHS) with encapsulated HRP-THI and HRP-Fc as distinguishable signal tags. A sandwich-type immunoassay was employed for the online detection of CEA and AFP by coupling a flow-through detection cell with an external magnet. The multiplexed method can pull antibodies bound to magnetic nanoparticles from one laminar flow path to another by applying a local magnetic field gradient, and selectively remove them from flowing biological fluids without any washing steps. Figure 12 shows as the assay involved the catalytic reduction of H_2_O_2_ at the various peak potentials in the presence of the corresponding mediators. The method enabled the simultaneous monitoring of AFP and CEA in a single run through wide working ranges of 0.01–200 ng·mL^−1^ for AFP and 0.01–80 ng·mL^−1^ for CEA [17].

Paper is an attractive substrate for the fabrication of microfluidic devices since it is a low cost and abundant material, disposable and biodegradable, and easy to manipulate and to be chemically modified [96]. Microfluidic paper-based analytical devices (μ-PADs) were firstly proposed by Whitesides et al. [97] combining the simplicity and portability of paper strip tests and the capacity of lab-on-a-chip devices for multiplexed analysis [98]. The reported configurations of electrochemical immunosensors using μ-PADs technology for the simultaneous determination of biomarkers are mainly based on patterned paper where small volumes of fluids run by capillary action. Moreover, they incorporate nanomaterials for enhancing the sensing probe immobilization as well as the sensitivity of detection. Furthermore, the origami approach has been demonstrated to be an adequate method for construct these configurations, existing various examples of multiplexed immunoassays using this technique. For example, a 3D microfluidic electrochemical origami immunosensor was developed for the simultaneous detection of CA125 and CA19-9 biomarkers. It utilized cuboid silver-modified paper working electrodes as sensor platform and Ag^+^ or Cu^2+^-coated nanoporous silver-chitosan as labels. Cuboid silver, acting as a model biocompatible nanomaterial with large surface area, high conductivity and absorption capability, was grown on the cellulose fibers surface from AgNPs seeds. The determination of both markers was carried out by SW voltammetric responses of metallic ions allowing dynamic linear ranges of over four orders of magnitude with detection limits down to 0.02 and 0.04 mU·mL^−1^ , respectively [99].

**Figure 13:**
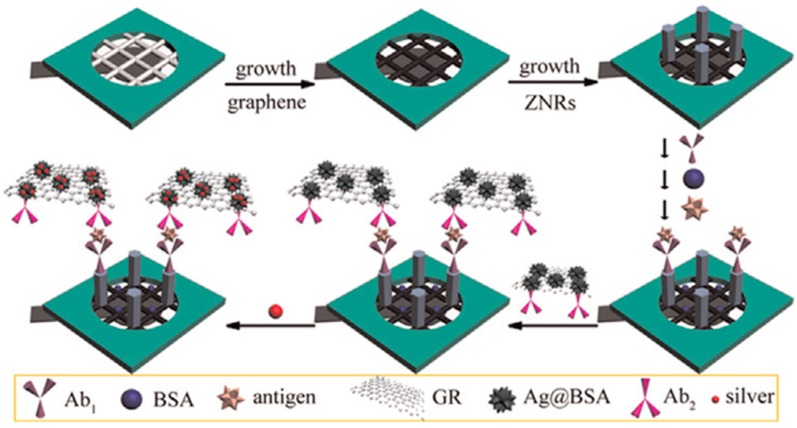
Fabrication process of the paper electrode-based immunosensor exemplified for one analyte. Reprinted from Ref. [100] with permission.

Sun et al. [100] prepared a microfluidic paper-based analytical device (μ-PAD) in which rGO was used to modify the cellulose fibers. ZnO nanorods, with a large surface-to-volume ratio and abundant sites for capturing biomolecules, were employed to immobilize the specific antibodies (Figure 13). Sandwich immunoassays were implemented for the detection of three target analytes, HCG, PSA and CEA, using composites of rGO and flower-like Ag@BSA nanoparticles, with a high electrocatalytic effect toward H_2_O_2_ reduction, as the signal labels conjugated with the secondary antibodies. After the immunocomplexes formation, the amperometric current was generated from the addition of H_2_O_2_ further amplified by the promoted deposition of silver. This method provided good linear relationships between the currents and the logarithm values of the analyte concentrations in the range of 0.002–120 mI UmL^−1^ for HCG, 0.001–110 ng·mL^−1^ for PSA, and 0.001–100 ng·mL^−1^ for CEA, and was successfully applied to the analysis of clinical serum samples.

Despite the social relevance and current demand, electrochemical multiplexed immunosensors have not yet been widely used for the detection of infectious diseases. Indeed only one method has been found in the literature regarding the simultaneous determination of antibodies against human immunodeficiency (HIV) and hepatitis C (HCV) viruses in serum using a paper-based immunosensing platform. It consisted of an electrochemical microfluidic paper-based array containing eight screen printed electrodes. To capture the target antibodies, the corresponding antigens were immobilized onto the working electrodes modified with 3-amino-propyl dimethyl-ethoxysilane (APDES) and glutaraldehyde. Once the immunocomplex was formed, IgG labeled with alkaline phosphatase was added, and the interaction was monitored by addition of *p*-aminophenylphosphate. The electrochemical detection was performed using amperometry, and the limits of detection achieved were of 300 and 750 pg·mL^−1^, for HIV and HCV, respectively. This immunosensing platform was applied to the analysis of serum samples with good results [101].

## 5. General Conclusions and Future Prospects

Multiplexed electrochemical immunosensors continue to evolve with improved sensitivity to develop new analytical strategies and applications for biomarkers determination. However, they still have a long way ahead before coming a competitive technology for development of point-of-care diagnostic testers. Most common approaches are prepared from platforms of a single electrode in barcode design, and applied to dual-analyte monitoring. However, only few devices have detected three or more markers. In this context, the design of new distinguishable species involving nanomaterials and/or electroactive probes which would produce more than two independent electrochemical signals, is a matter of concern. Related to cancer biomarkers, the possibility of measure panels of proteins is a desirable objective to provide faster and more accurate diagnostics. Although the number of multiplexed electrochemical immunosensors for these species has greatly increased in the last three years, however, the novel designs are usually applied to the same biomarkers, so that there are several immunosensors for the most common, AFP, CEA and PSA, and very few or none for other important protein biomarkers. Indeed the identification and clinical validation of new and reliable biomarkers signatures will be compulsory for these attractive multiplexing electrochemical biosensing platforms find wide applicability also for other relevant diseases apart from cancer such as inflammatory processes, cardiovascular, autoimmune and degenerative diseases. Moreover, since the biomarkers panel identified could comprise biomarkers with high differences in the threshold levels, additional efforts should be focused in developing strategies suitable to determine simultaneously biomarkers at very different concentration levels.

The overall data available in the literature on recent multiplexed immunosensing configurations demonstrate the adequate performance of label-free electrochemical immunosensors, where nanomaterials play a fundamental role, both as a mean for biomolecules immobilization and to take advantage of their catalytic activity for different processes. In addition, microfluidics continues to emerge as an attractive technology to achieve automation of immunoassay methods, decreasing the analysis times and paving the way for the development of POCs testing devices.

However, it is worth to mention also that despite the tremendous progress made these last years in the development and application on ultrasensitive immunosensing platforms for multiplexed determination of clinical biomarkers, none has crossed the technological valley of death to successful commercialization. Many times the validation of the electrochemical immunosensors developed is limited to doped biological samples such as blood, serum, saliva and urine or to a very limited number of real samples, which is not enough to ensure their reliable validation. In this sense, a better communication between the end users of the devices and the scientists who develop the methodologies and the availability of samples for large case studies will play major roles in the exhaustive validation of these devices. Clinical validation using minimally treated real samples and exhaustive comparison with other current methodologies will be required to ensure the transition from research laboratories to the market and for their implementation as a POC devices. Additional efforts to guarantee appropriate functionality after transportation and during storage will be crucial also to accelerate their commercialization as clinical diagnosis/prognosis routine tools.

Despite these formidable challenges to address, the attractive properties of these multiplexed electrochemical immunosensing devices make them extremely promising for improving the reliability and quickness of diagnostics and therapy monitoring, leading to more rapid clinical decision making and corresponding reductions in patient stress and healthcare costs. This will assured them a privileged place in the future clinical field.

## Figures and Tables

**Table 1:** Multiplexed electrochemical immunosensors involving electrode arrays.

Electrodes	Label	Analyte	Immunoassay	Technique	Linear Range	LOD	Sample	Ref.
Cancer biomarkers								
GA/Chit/SPAuEs (2WEs)	Strept/AuNPs/CNHs	AFP CEA	Sandwich-type; Ag deposition	LSASV	0.1–1000 pg·mL^−1^	0.024 pg·mL^−1^ AFP 0.032 pg·mL^−1^ CEA	serum	[7]
SPAuEs (4WEs)	AuNRs-HRP	phospho-p53_392_ phospho-p53_15_ phospho-p53_46_ total p53	Sandwich-type; detection of H_2_O_2_/THI	SWV	0.01–20 nM p53_392_ 0.05–20 nM p53_15_ 0.1–50 nM p53_46_ 0.05–20 nM total p53	5 pM p53_392_ 20 pM p53_15_ 30 pM p53_46_ 10 pM total p53	plasma	[8]
carboxylated-SPCEs (16WEs)	HRP-MWCNTs	PSA IL-8	Sandwich-type; detection of TMB	amperometry	5–4000 pg·mL^−1^ PSA 8–1000 pg·mL^−1^ IL-8	5 pg·mL^−1^ PSA 8 pg·mL^−1^ IL-8	serum	[18]
AuNPs film/PDMS (8 × 8 WEs)	HRP/AuNRs	PSA; PSMA; IL-6	Sandwich-type; detection of H_2_O_2_	amperometry	0.1–10 ng·mL^−1^ PSA 0.005–1 ng·mL^−1^ IL-6 0.8–400 PSMA	0.1 ng·mL^−1^ PSA 0.005 ng·mL^−1^ IL-6 0.8 ng·mL^−1^ PSMA	serum	[19]
G/SPCEs (3WEs)	M-PtNPs	CA125, CA153, CEA	Sandwich-type; detection of H_2_O_2_	DPV	0.05–20 U·mL^−1^ CA125; 0.008–24 U·mL^−1^ CA153; 0.02–20 24 U·mL^−1^ CEA	0.002 U·mL^−1^; 0.001 U·mL^−1^; 7.0 pg·mL^−1^	serum	[20]
Chit/SPCEs (2WEs)	AuNPs	CEA AFP	Sandwich-type; Ag deposition	LSASV	0.005–5.0 ng·mL^−1^	3.5 pg·mL^−1^ CEA 3.9 pg·mL^−1^ AFP	serum	[21]
Chit/SPCEs	Strept/AgNPs/MWCNTs	CEA AFP	Sandwich-type; Ag deposition	LSASV	0.1–5000 pg·mL^−1^	0.093 pg·mL^−1^ CEA 0.061 pg·mL^−1^AFP	serum	[22]
AuNPs/G/SPCEs (3WEs)	AP-AuCs/G	CA153, CA125, CEA	Sandwich-type; addition of 3-IP and Ag deposition	LSASV	0.005–50 U·mL^−1^ CA 153 0.001–100 U·mL^−1^ CA 125 0.004–200 U·mL^−1^ CEA	0.0015 U·mL^−1^ CA 153 0.00034 U·mL^−1^ CA 125 0.0012 U·mL^−1^ CEA	serum	[23]
SPCEs (2WEs)	Strept-HRP	ERα PR	Sandwich-type magnetoimmuno-sensor; detection of H_2_O_2_/HQ	amperometry	73–1500 pg·mL^−1^ (PR)	22 pg·mL^−1^ (PR)	cell lysates	[24]
Cardiac biomarkers								
CNF microE chip (3 × 3)	-	CRP, cTNI, Mb	Direct; detection of Fe(CN)_6_^4−^	DPV	-	-	serum	[25]
HOOC-MBs/SPCEs (2WEs)	HRP	NT-proBNP CRP	Competitive ( NT-pro BNP); sandwich-type (CRP); detection of H_2_O_2_ in presence of TMB	amperometry	2.5–504 ng·mL^−1^ NT-proBNP 2–100 ng·mL^−1^ CRP	0.47 ng·mL^−1^	serum	[26]
SiNW array	-	cTnT, CK-MM, CK-MB	Direct assay	Resistance changes caused by specific binding	1 pg·mL^−1^–10 ng·mL^−1^	1 pg·mL^−1^ (2 μL blood 45 min)	blood	[27]
Other clinical biomarkers								
HOOC-Phe-DWCNTs/SPCEs (2WEs)	poly-HRP-Strept	IL-1β TNF-α	Sandwich-type; detection of H_2_O_2_ in the presence of HQ	amperometry	0.5–100 pg·mL^−1^ IL-1β 1–200 pg·mL^−1^ TNF-α	0.38 pg·mL^−1^ IL-1β 0.85 pg·mL^−1^ TNF-α	serum saliva	[28]
Au microE chip (8WEs)	-	IL-1β, IL-10, IL-6	Direct; detection of Fe(CN)_6_^3−/4−^	EIS	1–15 pg·mL^−1^	-	-	[29]
rGO/SPCEs (2WEs)	Strept-AP	GHRL PYY	Sandwich-type; addition of 1-NPP	DPV	10^−3^–100 ng·mL^−1^ GHRL 10^−4^–10 ng·mL^−1^ PYY	1.0 pg·mL^−1^ GHRL 0.02 pg·mL^−1^ PYY	serum saliva	[30]
SPCEs (2WEs)	Strept-AP	cortisol ACTH	Sandwich-type; addition of 1-NPP	DPV	0.1–500 ng·mL^−1^ cortisol 5.0 × 10^−5^–0.1 ng·mL^−1^ ACTH	37 pg·mL^−1^ cortisol 0.04 pg·mL^−1^ ACTH	serum	[31]
AuNPs/MWCNTs/SPCEs (2WEs)	AP	IgA and IgG type AGA anti-tTG	Sandwich-type; addition of 3-IP and Ag deposition	cyclic-ASV	up to 100 U mL^−1^	2.45 U·mL^−1^ tTG IgA 2.95 U·mL^−1^ tTG IgG	serum	[32]

**Abbreviations:** AFP, alpha fetoprotein; AP, alkaline phosphatase; AuNRs, gold nanorods; BSA, bovine serum albumin; CA125, CA153, carbohydrate antigen 123 or 153; CEA, carcinoembryonic antigen; Chit, chitosan; CNF, carbon nanofiber; DPV, differential pulse voltammetry; EIS, electrochemical impedance spectrospocy; ERα, estrogen receptor α; G, graphene; GA, glutaraldehyde; GHRL, ghrelin; hCG, human chorionic gonadotropin; HRP, horse-raddish peroxidase; IL, interleukin; 3-IP, 3-indoxyl phosphate; ITO, indium tin oxide electrode; LSASV, linear sweep-anodinc stripping voltammetry; MPtNPs, mesoporous platinum nanoparticles; 1-NPP, 1-naphthylphosphate; PB, Prussian blue; PDMS, polydimethylsiloxane; PSA, prostate specific antigen; PSMA, prospate specific membrane antigen; PWE, paper working electrode; PYY, peptide YY; rGO, reduced graphene oxide; SPAuEs, screen-printed gold electrodes; SPCEs, screen-printed carbon electrodes; Strept, streptavidin; TB, toluidine blue; TCCRMSs, tryptophan and caffeic acid-based resin; THI, thionine;.TMB, 3,3’,5,5’-tetramethylbenzidine; TNF-α, tumor necrosis factor alfa; WE, working electrode; ZNRs, zinc oxide nanorods.

**Table 2:** Multiplexed electrochemical immunosensors using barcode configurations.

Electrode	Label	Analyte	Immunoassay	Technique	Linear Range	LOD	Sample	Ref.
Cancer biomarkers								
G/Au/GCE	THI (or Co or Fc or AQ)-Strept-Biotin-dsDNA/Strept-Biotin-Ab_2_/AuNPs/SiO_2_/Fe_3_O_4_	CEA, CA125, PSA, AFP	Sandwich-type; direct detection after conjugation	DPV	0.2–600 pg·mL^−1^ CEA; 0.2–1000 pg·mL^−1^ CA125; 0.2–800 pg·mL^−1^ PSA; 0.2–800 pg·mL^−1^ AFP	48 fg·mL^−1^ CEA; 77 fg·mL^−1^ CA125; 60 fg·mL^−1^ PSA; 62 fg·mL^−1^AFP	-	[6]
AuNPs/GCE	PVP/GS/PtPdNPs/HRP/AQ PVP/GS/PtPdNPs/HRP/THI	AFP, APT; DCP, AFP-L3, γ-GT, AFU	Sandwich-type; addition of H_2_O_2_	DPV	0.025–5.0 ng·mL^−1^ AFP; 0.024–9.6 ng·mL^−1^ APT; 0.032–3.2 UL^−1^ DCP; 0.024–2.4 ng·mL^−1^ AFP-L3; 1.0–9.5UL^−1^ γ-GT; 1.2–9.0 UL^−1^ AFU	0.008 ng·mL^−1^ AFP; 0.0082 ng·mL^−1^ APT; 0.01UL^−1^ DCP; 0.008 ng·mL^−1^ AFP-L3; 0.33 UL^−1^ γ-GT; 0.4 UL^−1^ AFU	serum	[12]
GCE (Hg)	PAMAM-CdS (or ZnS, or PbS)	CA125 CA15-3 CA19-9	Sandwich-type; direct detection of Cd, Zn and Ag	SWASV	0.01–50 U·mL^−1^	0.005 U·mL^−1^	serum	[13]
IL/rGO/GCE	PtNPs-Cu^2+^ PtNPs-Cd^2+^	AFP CEA	Sandwich-type; reduction of metal ions	DPV	0.05–200 ng·mL^−1^	0.05 ng·mL^−1^ AFP 0.002 ng·mL^−1^ CEA	serum	[14]
AuNPs/Chit/GCE	CGS-PB; CGS-TB	CEA; AFP	Sandwich-type; direct detection	DPV	0.5–60 ng·mL^−1^	0.1 ng·mL^−1^ (CEA) 0.05 ng·mL^−1^ (AFP)	serum	[33]
AuNPs/Chit/GCE	Chit/AuNPs/PB; Chit/AuNPs/Fc	CEA; AFP	Sandwich-type; direct detection	DPV	0.05–100 ng·mL^−1^	0.02 ng·mL^−1^ (CEA) 0.03 ng·mL^−1^ (AFP)	serum	[34]
HAG/PANI/rGO/GCE	AuNPs/rGO/PB; AuNPs/rGO/PDDA/THI	CEA; AFP	Sandwich-type; direct detection	DPV	0.6–80 ng·mL^−1^	0.12 ng·mL^−1^ (CEA) 0.08 ng·mL^−1^ (AFP)	serum	[35]
AuNPs/GCE	Fc/HRP THI/HRP	CEA AFP	Direct assay after addition of H_2_O_2_	DPV	0.01–50 ng·mL^−1^	0.01 ng·mL^−1^	serum	[36]
AuNPs/G/GCE	THI-HRP-NPG-MnO_2_ Fc-HRP-NPG-MnO_2_	CA-15-3 CA-125	Sandwich-type; addition of H_2_O_2_	SWV	0.01–50 U·mL^−1^	3.5 mU·mL^−1^ (CA-153) 4.1 mU·mL^−1^(CA-125)	serum	[37]
PDPMT-Cl-Chit/GCE	THI-Fe_3_O_4_NPs Fca-Fe_3_O_4_NPs	SCC-Ag CEA	Sandwich; direct detection	DPV	0.01–10 ng·mL^−1^	4 pg·mL^−1^ (SCC-Ag) 5 pg·mL^−1^ (CEA)	serum	[38]
CD/G/GCE	CD/Fc/G CD/THI/G	CEA AFP	Sandwich-type with HRP-Ab_2_ and H_2_O_2_ detection	DPV	0.003–40 ng·mL^−1^ CEA 0.001–60 ng mL^−1^ AFP	0.8 pg·mL^−1^ CEA 0.5 pg·mL^−1^ AFP	serum	[39]
rGO/THI (or PB)/AuNPs/ITO	-	CEA AFP	Direct detection of of THI or PB	SWV	0.01–300 ng·mL^−1^	0.650 pg·mL^−1^ CEA 0.885 pg·mL^−1^ AFP	serum	[40]
TB (orPB)/AuNPs/TCCRMSs/FTO	-	CEA NSE	Direct; detection of TB or PB	SWV	2–25 ng·mL^−1^	0.11 ng·mL^−1^ CEA 0.08 ng·mL^−1^ NSE	serum	[41]
HOOC-MBs/SPCE	ACTP/AuNPs ATLP/AuNPs	p53^15^, p53^392^	Sandwich-type; detection of metals	SWV	1–20 ng·mL^−1^ (p53^15^) 0.5–20 ng·mL^−1^ (p53^392^)	0.5 ng·mL^−1^ (p53^15^) 0.2 ng·mL^−1^ (p53^392^)	serum	[42]
GCE (Bi)	G/AuNPs-r-Apo-Cd G/AuNPs-r-Apo-Pb	AFP CEA	Sandwich-type; detection of metals	SWASV	0.001–5 ng·mL^−1^	0.3 pg·mL^−1^ AFP; 0.35 pg·mL^−1^ CEA	serum	[43]
rGO	CdSeTe@CdS Ag NCs	Bcl-2 Bax	Sandwich-type; direct detection of Cd and Ag	SWASV	1–250 ng·mL^−1^	0.5 fmol	leukemiaK562 cells	[44]
Chit/MWCNTs/GCE	OMC-Zn OMC-Cd	AFP HER-2	Sandwich-type; oxidation of metal ions	DPV	0.001–150 ng·mL^−1^	0.6 pg·mL^−1^ AFP 0.35 pg·mL^−1^ HER-2	serum	[45]
GCE	Envision^TM^-CdS (or PbS or AuNPs)	AFP CEA CA19-9	Sandwich-type; detection of metals	DPASV DPCSV	0.001–50 ng·mL^−1^ AFP; CEA; 0.005–100 ng·mL^−1^ CA19-9	0.02 pg·mL^−1^ AFP; 0.05 pg·mL^−1^ CEA; 0.3 pg·mL^−1^ CA19-9	serum	[46]
Glu/Chit/AuNPs/AuE	BSA/AuNPs-Pb^2+^ BSA/AuNPs-Cd^2+^	CEA AFP	Sandwich-type; reduction of metal ions	DPV	0.01–50 ng·mL^−1^	4.6 pg·mL^−1^ CEA 3.0 pg·mL^−1^ AFP	serum	[47]
AuNPs/AuE	AuNPs@MWCNTs-Pb^2+^ AuNPs@MWCNTs-Cd^2+^	CEA AFP	Sandwich-type; reduction of metal ions	SWV	0.01–60 ng·mL^−1^	3.0 pg·mL^−1^ CEA 4.5 pg·mL^−1^ AFP	serum	[48]
AuNPs/rGO/Chit	AgNPs/THI/CNSs AgNPs/CNSs	CEA AFP	Sandwich-type; direct detection	DPV	0.01–80 ng·mL^−1^	2.8 pg·mL^−1^ CEA 3.5 pg·mL^−1^ AFP	serum	[49]
IL/rGO/PSS/GCE	CGN-THI CGN-DAP CGN-Cd^2+^	CEA PSA AFP	Sandwich-type; direct detection	SWV	0.01–100 ng·mL^−1^	2.7 pg·mL^−1^ CEA 4.8 pg·mL^−1^ PSA 3.1 pg·mL^−1^ AFP	serum	[50]
AuNPs/AuE	TiO_2_/Nf- Co(dcbpy)_3_^2+^ TiO_2_/Nf- MB	CA15-3 CA19-9	Sandwich-type; direct detection	DPV	5–100 U·mL^−1^ CA15-3 1–100 U·mL^−1^ CA19-9	0.3 UmL^−1^ CA15-3 1.6 UmL^−1^ CA19-9	serum	[51]
AuNPs/PAPT/GCE	poly(VFc-ATP) AuNPsPOPD/AuNPs	AFP CEA	Sandwich-type; direct detection	DPV	0.01–100 ng·mL^−1^	0.003 ng·mL^−1^ AFP 0.006 ng·mL^−1^ CEA	serum	[52]
AuNPs/G/GCE	PS-Cd^2+^ PS-Fc	IL-6 IL-17	Sandwich-type; direct detection	SWV	1–1000 pg·mL^−1^ IL-6 2–1000 pg·mL^−1^ IL-17	0.5 pg·mL^−1^ 1 pg	serum	[53]
Protein A/Nafion/GCE	GS/AuNPs-THI (or Cobpy)_3_^3+^ or Fc)	AFP CEA SS2	Sandwich.type; direct detection	DPV	0.016–50 ng·mL^−1^ AFP 0.010–50 ng·mL^−1^CEA 0.012–50 ng·mL^−1^ AFP	5.4 pg·mL^−1^ AFP 2.8 pg·mL^−1^ CEA 4.2 pg·mL^−1^ AFP	-	[54]
AuNPs/PEI/PTCA/GCE	Au@PBNPs/O-GS-Strept-AP; Au@NiNPs/O-GS-Strept-AP	fPSA PSA	Sandwich-type; AA-P addition	DPV	0.02–10 ng·mL^−1^ fPSA 0.01–50 ng·mL^−^^1^ PSA	6.7 pg·mL^−^^1^ 3.4 pg·mL^−^^1^	serum	[55]
MWCNTs/GCE	AA liposome UA liposome	NSE ProGRP	Sandwich-type, direct detection	LSV	50–1000 pg·mL^−1^	5.0 pg·mL^−1^ NSE 100 pg·mL^−1^ ProGRP	serum	[56]
AuNPs/IL/rGO/GCE	Cd (or Pb or Cu) AlgNBs	AFP CEA PSA	Sandwich-type; direct detection	DPV	0.01–100 ng·mL^−1^	0.01 ng·mL^−1^AFP 0.0086 ng·mL^−1^CEA 0.0075 ng·mL^−1^PSA	serum	[57]
GCE (Bi)	PLL/AuNPs/Cd-Apo (or Pb-Apo)	AFP CEA	Sandwich-type; direct detection	ASSWV	0.01–50 ng·mL^−1^	4 pg·mL^−1^	serum	[58]
Chit/AuNPs/GCE	GA/Chit/pAA NSs-Cu^2+^ (or Cd^2+^ or Zn^2+^)	CEA CA19-9 CA125 CA242	Sandwich-type; direct detection	SWV	0.1–100 ng·mL^−1^ CEA 1–150 U·mL^−1^ CA 19-9, CA125,CA242	0.02 ng·mL^−1^ CEA 0.4 U·mL^−1^ CA19-9 0.3 U·mL^−1^ CA125 0.4 U·mL^−1^ CA242	serum	[59]

**Abbreviations:** AA, ascorbic acid; AA-P, ascorbic acid 2-phosphate; ACTP, apoferritin templated cadmium phosphates; AFP, alpha fetoprotein; AFP-L3, lens culinaris (LCA)-reactive fraction of AFP; AFU, alpha-L-fucosidase; AgNCs, silver nanoclusters; Alg, alginate; Apo, apoferritin; APT, abnormal prothrombin; ATLP, apoferritin templated lead phosphates; AU, uric acid; Bax, Bcl-2 associated X protein; Bcl, B-cell lymphoma; CEA, Chit, chitosan; CGN, carbon-gold nanocomposite; CGS, carboxyl graphene nanosheet; CNS, carbon nanospheres; DCP, des-c-carboxy prothrombin; Fc. ferrocene; fPSA, free-prostate specific antigen; FTO, fluorine-doped tin oxide; G, graphene; γ-GT, γ-glutamyltranspeptidase; GCE, glassy carbon electrode; O-GS, onion-like graphene sheet; HER-2, human epidermal growth factor receptor type-2; HRP, horse-raddish peroxidase; IL, ionic liquid; NPG, nanoporous gold; NSE, neuron-specific enolase; OMC, ordered mesoporous carbon; pAA, polyacrylic acid; PATP, poly(2-aminothiophenol); PB, Prussian blue; PDPMT-Cl, poly(3-(1,1-o-dimethyl-4-piperidine-methylene)thiophene-2,5-diylchloride); PEI, polyethyleneimine; PLL, poly-L-lysine; POPD, poly(o-phenylenediamine); ProGRP, pro-gastrin-releasing peptide; PTCA, 3,4,9,10-perylenetetracarboxylic acid; SS2, streptococcus suis serotype 2; TB, toluidine blue; TCCMRSs, tryptophan and caffeic acid-based resin microspheres; TB, toluidine blue; THI, thionine; UA, uric acid; VFc-ATP, vinyl ferrocene-2-aminothiophenol.
